# Three-Axis Coil Probe Dimensions and Uncertainties During Measurement of Magnetic Fields from Appliances

**DOI:** 10.6028/jres.099.020

**Published:** 1994

**Authors:** Martin Misakian, Charles Fenimore

**Affiliations:** National Institute of Standards and Technology, Gaithcrsburg, MD 20899-0001

**Keywords:** appliance, coil probe, magnetic field, measurement, measurement uncertainty, power frequency

## Abstract

Comparisons are made between the average magnetic flux density for a three-axis circular coil probe and the flux density at the center of the probe. The results, which are determined assuming a dipole magnetic field, provide information on the uncertainty associated with measurements of magnetic fields from some electrical appliances and other electrical equipment. The present investigation extends an earlier treatment of the problem, which did not consider all orientations of the probe. A more comprehensive examination of the problem leaves unchanged the conclusions reached previously.

## 1. Introduction

This paper reconsiders a problem related to the measurement of power frequency magnetic fields from electrical appliances using three-axis circular coil probes. Specifically, it reexamines the differences between the average magnetic flux density as determined using a magnetic field meter with a three-axis circular coil probe and the magnetic flux density at the center of the probe, *B*_0_, assuming the field is produced by a small loop of alternating current, i.e., a magnetic dipole. The “average” arises as a consequence of the averaging effects of the coil probes over their cross sectional areas when placed in a nonuniform magnetic field. The differences between the average magnetic field and *B*_0_ can be regarded as measurement errors because the center of the probe is normally considered the measurement location. The magnetic dipole field is chosen as the relevant field because its geometry provides a good approximation of the magnetic field produced by many electrical appliances [1].

The average magnetic flux density measured by a three-axis magnetic field meter, *B*_av3_, is also referred to as the resultant magnetic field and is defined as [2]

$$B_{\mathrm{av}3}=\sqrt{B_{1}^{2}+B_{2}^{2}+B_{3}^{2}},$$ (1)

where *B*_1_, *B*_2_, and *B*_3_ are average root-mean-square (rms) magnetic field components determined by each of three orthogonally oriented coil probes.

Differences between *B*_av3_ and *B*_0_ are calculated as a function *of r/a* where *r* is the distance between the magnetic dipole and the center of the probe, and *a* is the radius of the three-axis probe. In addition, differences between *B*_av3_ and *B*_0_ are examined for different orientations of the magnetic dipole and rotations of the three-axis probe. Because the relative orientation of the dipole and three-axis probe is not known during most measurement situations, there is a distribution of possible differences between *B*_av3_ and *B*_0_, and these differences collectively represent a source of measurement uncertainty for a given *r/a.* What will be of interest in this paper is the largest difference that occurs between *B*_av3_ and *B*_0_ as a function of *r/a* (for all possible orientations of the dipole). This largest difference is designated Δ*B*_max3_.

This investigation extends an earlier treatment of the problem which considered different orientations of the dipole, but not all possible orientations of the three-axis probe [3]. The maximum difference between B_av3_ and *B*_0_, Δ*B*_max3_, is found by a numerical search during which *B*_av3_ is determined by numerical integration. The major advance over the earlier study is the development of an expression giving the average magnetic flux density for a circular coil probe for any position and orientation of the probe in the dipole magnetic field. This development allows the search for Δ*B*_max3_ to consider “all” possible rotations of the three-axis probe. The extended search is shown to leave unchanged the values of Δ*B*_max3_ that were determined by the earlier treatment.

## 2. Expression for Average Magnetic Field

In the derivation given below, it is assumed that the cross sectional areas of the wire in the coil probes and the opposing magnetic fields produced by currents induced in the probes are negligible. We also assume that the three orthogonally oriented coils of the three-axis probe have circular cross sections of equal area. These assumptions either can be met in practice or can be taken into account by a calibration process.

**Fig. 1:**
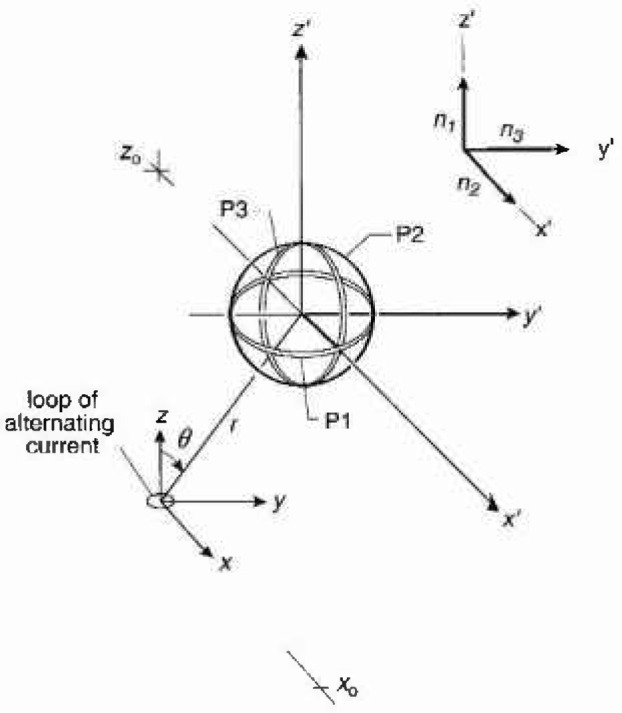
Three-axis magnetic field probe with its center at *x=x*_0_, *y* = 0, and z *=z*_0_. A small current loop producing a dipole magnetic field is located at the origin of the unprimed coordinate system. The unit vectors ***n***_1_, ***n***_2_, and **n**_3_ arc normal to the areas of probes P1, P2, and P3, respectively. Changes in the angle *θ* correspond to varying the orientation of the dipole with respect to the probe.

The average magnetic flux density, *B*_av_, for a single circular coil probe with cross sectional area *A* is given by

$$B_{\mathrm{av}}=\frac{1}{A}\iint_{A}B\cdot n\;dA,$$ (2)

where d*A* is an element of probe area, ***n*** is a unit vector perpendicular to *A*, and ***B*** is the magnetic flux density. In spherical coordinates, the magnetic flux density for a small current loop of radius *b* is [4]

$$B=\frac{\mu_{0}Ib^{2}}{2r^{3}}\cos\;\theta u_{r}+\frac{\mu_{0}Ib^{2}}{4r^{3}}\sin\;\theta u_{0},$$ (3)

where μ_0_ is the permeability of vacuum, *I* is the alternating current, and ***u****_r_* and ***u****_θ_* are unit vectors in the directions of increasing *r* and *θ*, respectively. The assumption is made that *b*«*r*, and the sinusoidal time dependence of the field has been suppressed. The value of *B*_0_ is given by the magnitude of *B* [Eq. (3)]. Figure 1 shows the spherical coordinates *r* and *θ*, a small current loop at the origin of the coordinate system, and a sketch of a three-axis probe. The center of the probe coincides with the origin of the prime coordinate system *x′, y′*, and z′. The coil probes are labelled P1, P2, and P3, have unit normal vectors ***n***_1_, ***n***_2_, and ***n***_3_, respectively, and are shown in Figure 1 (inset) for illustrative purposes as being in the directions of prime coordinates. The orientation of the magnetic dipole with respect to the position of the probe is characterized by the angle *θ.*

For our purposes, it is convenient to express ***B*** in terms of Cartesian coordinates. The magnetic flux density is then [2]

$$B=i\frac{3Cxz}{2r^{5}}+j\frac{3Cyz}{2r^{5}}+k\frac{C}{2r^{3}}\left(\frac{3z^{2}}{r^{2}}-1\right),$$ (4)

where *r* = [*x*^2^*+y*^2^*+z*^2^]^1/2^, ***i***, ***j***, and ***k*** are unit vectors for the Cartesian coordinates, and *C* is the constant *μ*_0_*Ib*^2^/2.

The goal is to develop an expression for *B*_av_ at an arbitrary point which can be evaluated for any orientation of the coil probe. The value of *B*_av3_ can then be found by combining the rms values of S_av_ from three orthogonal directions according to Eq. (1). The approach described below for obtaining the desired expression for *B*_av_ is to transform the problem into the coordinate system of the coil probe. In this coordinate system, the unit vector normal to the plane of the coil coincides with the “z-axis”, ***B*** is expressed in terms of the probe coordinates, and the integration over the area of the circular coil probe is carried out numerically in polar coordinates.

**Fig. 2:**
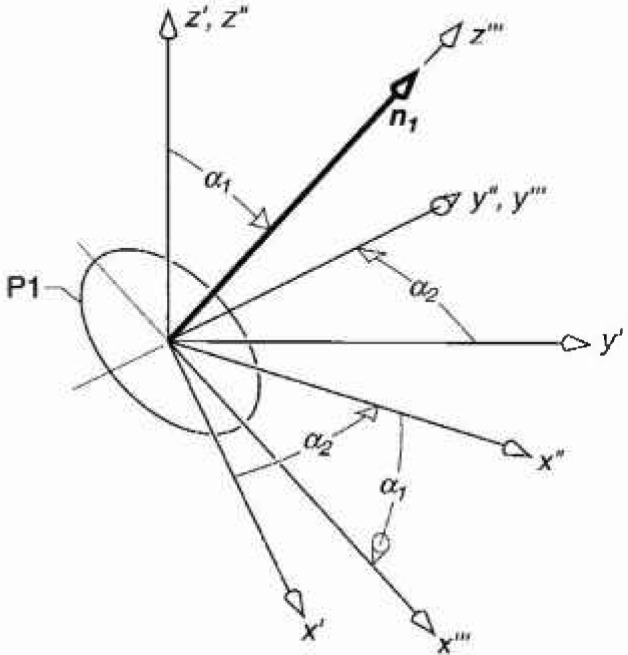
(a) Geometry of unit vector ***n***_1_ and coordinates after rotation of the prime coordinates through angle *α*_2_ about the z′-axis and after rotation of double-prime coordinates through angle *α*_1_ about *y*″-axis.

We begin by considering, without loss of generality, a three-axis coil probe with its center at *x=x*_0_, *y* = 0, and *z = z*_0_, where *x*_0_ *= r* sin*θ* and *z*_0_*=r* cos*θ* (Fig. 1). We then focus on coil probe P1 and its unit vector ***n***_1_ after it is rotated through angles *α*_1_ and *α*_2_ with respect to the prime coordinate system as shown in Fig. 2. The unit vectors ***n***_2_ and ***n***_3_ will also change in orientation to maintain their orthogonal relationship, but are not shown for purposes of clarity. In the prime coordinate system, ***n***_1_ is given by

$$n_{1}=i\;\sin\alpha_{1}\;\cos\alpha_{2}+j\sin\alpha_{1}\sin\alpha_{2}+k\cos\alpha_{1}.$$ (5)

By examination,

$$\begin{array}{l} n_{2}=i\sin(\alpha_{1}+90{}^{\circ})\cos\alpha_{2}+j\sin(\alpha_{1}+90{}^{\circ})\sin\alpha_{2}+k\cos(\alpha_{1}+90{}^{\circ})\\ =i\cos\alpha_{1}\cos\alpha_{2}+j\cos\alpha_{1}\sin\alpha_{2}-k\sin\alpha_{1}. \end{array}$$ (6)

The remaining unit vector, ***n***_3_, is given by

$$\begin{array}{l} n_{3}=n_{1}\times n_{2}\\ =-i\sin\alpha_{2}+j\cos\alpha_{2}, \end{array}$$ (7)

but it also can be determined by examination, i.e., *α*_1_ is replaced by 90° and *α*_2_ is replaced by *α*_2_ + 90° in Eq. (5). For this case, ***n***_3_ is constrained to lie in the *x′-y′* plane. Later this constraint is removed.

The coordinate system of the probe is reached by the following transformations:

- translation of the origin to the origin of the prime coordinates shown in Fig. 1,
- rotation of the prime coordinates through angle *α*_2_ about the z*′*-axis, yielding the double-prime coordinates *x*″, *y*″, *z″*, as shown in Fig. 2, and
- rotation of the double-prime coordinates through angle *α*_1_ about the *y*″-axis yielding the triple-prime coordinates *x‴, y*‴, *z‴* (Fig. 2). In the triple-prime coordinate system, the normal vector, ***n***_1_, given by Eq. (5), is along the *z‴*-axis as desired.

Transformation (i) is given by

$$\begin{array}{l} x=x_{0}+x^{\prime }\\ y=y^{\prime }\\ z=z_{0}+z^{\prime }. \end{array}$$ (8)

The first rotation of coordinates (ii) is given by

$$\begin{array}{l} x^{\prime }=x^{\prime \prime }\cos\alpha_{2}-y^{\prime \prime }\sin\alpha_{2}\\ y^{\prime }=x^{\prime \prime }\sin\alpha_{2}+y^{\prime \prime }\cos\alpha_{2}\\ z^{\prime }=z^{\prime \prime }, \end{array}$$ (9)

and the second rotation (iii) is given by

$$\begin{array}{l} z^{\prime \prime }=z^{\prime \prime \prime }\cos\alpha_{1}-x^{\prime \prime \prime }\sin\alpha_{1}\\ x^{\prime \prime }=z^{\prime \prime \prime }\sin\alpha_{1}+x^{\prime \prime \prime }\cos\alpha_{1}\\ y^{\prime \prime }=y^{\prime \prime \prime }. \end{array}$$ (10)

From Eqs. (8)–(10), we have

$$\begin{array}{l} x=x_{0}+(z^{\prime \prime \prime }\sin\alpha_{1}+x^{\prime \prime \prime }\cos\alpha_{1})\cos\alpha_{2}-y^{\prime \prime \prime }\sin\alpha_{2}\\ y=(z^{\prime \prime \prime }\sin\alpha_{1}+x^{\prime \prime \prime }\cos\alpha_{1})\sin\alpha_{2}+y^{\prime \prime \prime }\cos\alpha_{2}\\ z=z_{0}+z^{\prime \prime \prime }\cos\alpha_{1}-x^{\prime \prime \prime }\sin\alpha_{1}, \end{array}$$ (11)

which, when substituted into Eq. (4), expresses ***B*** in terms of the probe coordinates. A simplification of Eq. (11) is had by noting that the integration over the area of the probe [Eq. (2)] occurs in the *x‴-y‴* plane, i.e., z‴=0. Contributions to *B*_av3_ from each of the coil probes is found by using the appropriate normal vector [Eqs. (5)–(7)] during the integrations. As noted above, the integration is carried out numerically (using a double Simpson’s Rule) in polar coordinates, i.e.,

$$\begin{array}{l} x^{\prime \prime \prime }=\rho \;\cos\psi ,\;0⩽\rho ⩽\alpha ,\;0⩽\psi <2\pi \\ y^{\prime \prime \prime }=\rho \;\sin\psi ,\\ dA=dx^{\prime \prime \prime }dy^{\prime \prime \prime }=\rho \;d\rho \;d\psi . \end{array}$$ (12)

The accuracy of the numerical integrations was checked by increasing the number of divisions between the limits of integration for *ρ* and *ψ.* The results reported below were not affected by further refinements of the intervals used during the integrations.

In the search for Δ*B*_max3_, it will be necessary to perform rotations of the three-axis probe about the z‴-axis or ***n***_1_ direction (see Search Protocol below), i.e., the unit vectors ***n***_2_ and ***n***_3_ for probes P2 and P3 are rotated about ***n***_1_. This removes the constraint noted earlier that ***n***_3_ lies in the *x′-y′* plane. Because the integrand for *B*_av_ is in terms of the *α* angles, relationships must be found between the angle of rotation about the z‴-axis, designated as *ϕ*, and the *α* values that appear in the integrands for P2 and P3. These relationships are found by examining the unit vectors for the probes ***n***_2_ and ***n***_3_ as they rotate about the *z*‴-axis or ***n***_1_ direction.

**Fig. 3:**
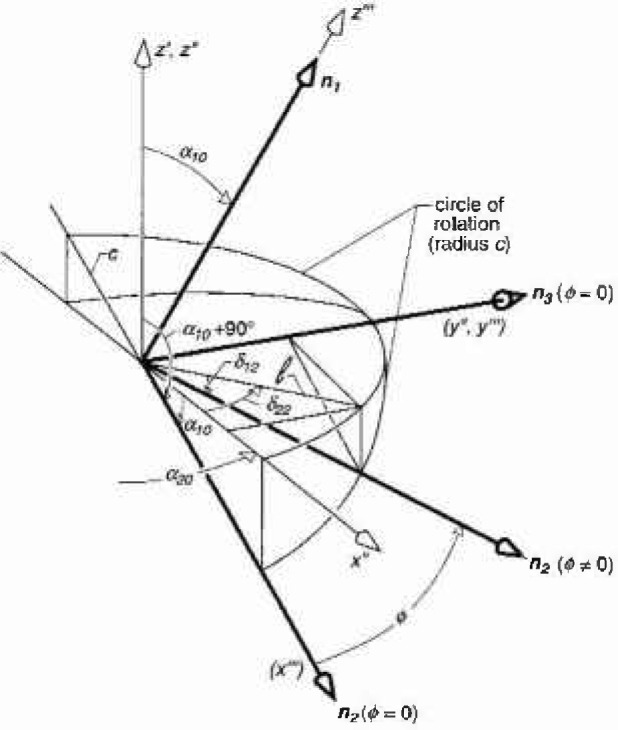
Geometry of coordinates and unit vectors after unit vector ***n***_2_ is rotated *ϕ* degrees about ***n***_1_, or z‴-axis. The rotation of ***n***_3_ is not shown for purposes of clarity (see Fig. 4).

**Fig. 4:**
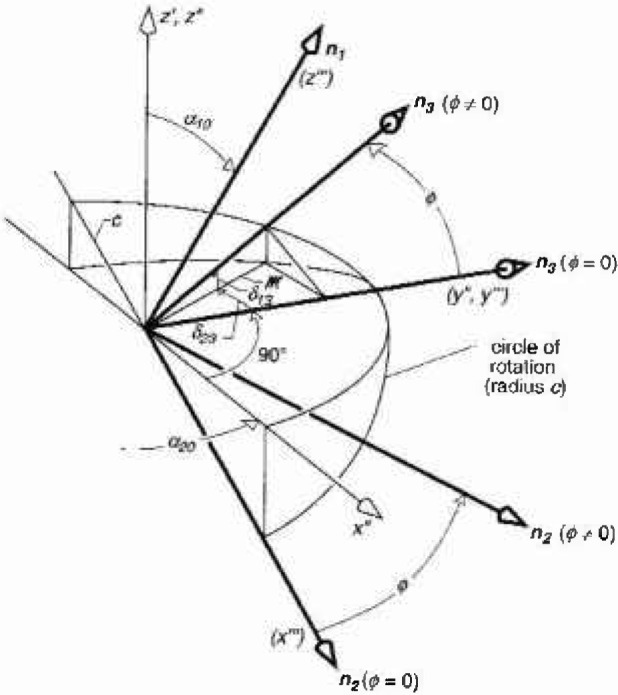
Geometry of coordinates and unit vectors after unit vectors ***n***_3_ and ***n***_2_ are rotated through angle *ϕ* about ***n***_1_ or *z*‴-axis.

In Figs. 3 and 4, consider ***n***_1_ in a direction characterized by the angles *α*_10_ and *α*_20_, i.e.,

$$n_{1}=i\sin\alpha_{10}\cos\alpha_{20}+j\sin\alpha_{10}\sin\alpha_{20}+k\cos\alpha_{10}.$$ (13)

From Eqs. (6) and (7), we have

$$n_{2}=i\sin(\alpha_{10}+90{}^{\circ})\cos\alpha_{20}+j\sin(\alpha_{10}+90{}^{\circ})\sin\alpha_{20}+k\cos(\alpha_{10}+90{}^{\circ}),$$ (14)

and

$$n_{3}=i\sin(90{}^{\circ})\cos(\alpha_{20}+90{}^{\circ})+j\sin(90{}^{\circ})\sin(\alpha_{20}+90{}^{\circ})+k\cos(90{}^{\circ}).$$ (15)

The trigonometric expressions in Eqs. (14) and (15) are not simplified in order to aid the reader in seeing the relationships between the three unit vectors.

Following a counterclockwise rotation of *ϕ* degrees about the z‴-axis, the *α*_2_’s will increase in value and the *α*_1_’s will decrease in value in the expressions for ***n***_2_ and ***n***_3_. These changes also occur in the expression for the magnetic flux density ***B***.

After a rotation of *ϕ* degrees, a line along the unit vector ***n***_2_ will intersect the circle of rotation in the*x‴-y‴* plane at a point given by (Fig. 3)

$$\begin{array}{l} x^{\prime \prime \prime }=c\;\cos\phi \\ y^{\prime \prime \prime }=c\;\sin\phi \\ z^{\prime \prime \prime }=0, \end{array}$$ (16)

where the radius for the rotation has been arbitrarily taken to be some constant *c*. From Eqs. (10) and (16), the same point in the double prime coordinate system is

$$\begin{array}{l} z\prime \prime =-x^{\prime \prime \prime }\sin\alpha_{10}=-c\;\cos\phi \sin\alpha_{10}\\ x\prime \prime =x^{\prime \prime \prime }\cos\alpha_{10}=c\;\cos\phi \cos\alpha_{10}\\ y\prime \prime =y^{\prime \prime \prime }=c\sin\phi . \end{array}$$ (17)

The increment to *α*_20_ for unit vector *n*_2_ after rotation *ϕ*, *δ*_22_ (Fig. 3), can be found from the expression for its tangent, i.e.,

$$\begin{array}{l} \tan\;\delta_{22}=\frac{y\prime \prime }{x\prime \prime }=\frac{\tan\phi }{\cos\alpha_{10}}\\ \delta_{22}=\tan^{-1}\left(\frac{\tan\phi }{\cos\alpha_{10}}\right). \end{array}$$ (18)

Prior to the rotation, the angle with respect to the *z*′-axis for the unit vector ***n***_2_ is given by *α*_10_ + 90° (Fig. 3). After the rotation, the corresponding angle will be *δ*_12_ + 90° where *δ*_12_ < *α*_10_. The value of *δ*_12_ is found by noting that after the rotation *ϕ*, the line *l* from the origin to the projection of the circle of rotation onto the *x″-y″* plane is given by

$$l=\sqrt{{\left(x\prime \prime \right)}^{2}+{\left(y\prime \prime \right)}^{2}},$$ (19)

and that the tangent for *δ*_12_ is just |*z*″/*l*| (Fig. 3). From Eqs. (17) and (19),

$$\delta_{12}=\tan^{-1}\left|\frac{z\prime \prime }{l}\right|=\tan^{-1}\left(\frac{\cos\phi \sin\alpha_{10}}{\sqrt{\cos^{2}\phi \cos^{2}\alpha_{10}+\sin^{2}\phi }}\right).$$ (20)

Thus, following a rotation of *ϕ* degrees about ***n***_1_, *α*_20_ and *α*_10_ + 90° will be replaced by *α*_20_ + *δ*_22_ and *δ*_12_ + 90°, respectively, in the expressions for ***n***_2_ and ***B*** during the calculation of *B*_av_ for probe P2.

The *α* values for ***n***_3_ can be determined with a similar analysis. Following a rotation of *ϕ* degrees about the z‴-axis (Fig. 4), a line along the unit vector ***n***_3_ will intersect the circle of rotation in the *x*‴-*y*‴ plane at the point

$$\begin{array}{l} x^{\prime \prime \prime }=-c\;\sin\phi \\ y^{\prime \prime \prime }=c\;\cos\phi \\ z^{\prime \prime \prime }=0. \end{array}$$ (21)

From Eqs. (10) and (21), the same point in the double prime coordinate system is

$$\begin{array}{l} z\prime \prime =c\;\sin\phi \sin\alpha_{10}\\ x\prime \prime =-c\;\sin\phi \cos\alpha_{10}\\ y\prime \prime =c\;\cos\phi . \end{array}$$ (22)

Following rotation *ϕ*, the angle *α*_20_ + 90° for ***n***_3_ [Eq. (15)] will increase by an amount *δ*_23_ as shown in Fig. 4. The increment, *δ*_23_, can be found from the expression for the absolute value of its tangent, i.e.,

$$\begin{array}{l} \tan\;\delta_{23}=\left|\frac{x\prime \prime }{y\prime \prime }\right|=\tan\phi \cos\alpha_{10},\\ \;\delta_{23}=\tan^{-1}\left(\tan\phi \cos\alpha_{10}\right). \end{array}$$ (23)

Prior to the rotation, the unit vector ***n***_3_ makes an angle of 90° with respect to the z′-axis (Fig. 4). After the rotation, this angle will decrease by an amount *δ*_13_. The value of *δ*_13_ is found by noting that after the rotation *ϕ*, the line *m* from the origin to the projection of the circle of rotation onto the *x″-y″* plane is given by

$$m=\sqrt{{\left(x\prime \prime \right)}^{2}+{\left(y\prime \prime \right)}^{2}},$$ (24)

and that the tangent for *δ*_13_ is just *z″/m* (Fig. 4).

From Eqs. (22) and (24) we have

$$\begin{array}{l} \tan\delta_{13}=\frac{z\prime \prime }{m}=\frac{\sin\phi \sin\alpha_{10}}{\sqrt{\sin^{2}\phi \cos^{2}\alpha_{10}+\cos^{2}\phi }}\\ \delta_{13}=\tan^{-1}\left(\frac{\sin\phi \sin\alpha_{10}}{\sqrt{\sin^{2}\phi \cos^{2}\alpha_{10}+\cos^{2}\phi }}\right). \end{array}$$ (25)

Thus, following a rotation of *ϕ* degrees about ***n***_1_, α_20_ + 90° and 90° are replaced by α_20_ + 90° + *δ*_23_ and 90°−*δ*_13_, respectively in the expressions for ***n***_3_ [Eq. (15)] and ***B*** during calculation of *B*_av_ for coil probe P3.

## 3. Search Protocol

The search for the largest difference between *B*_av3_ and *B*_0_, Δ*B*_max3_, for a given distance *r* from the dipole proceeds as follows:

- For a fixed distance *τ* away from the dipole, and with *θ = α*_1_ = *α*_2_ = 0, the three-axis probe is rotated about the *z*‴-axis or ***n***_1_ direction in 2° steps (i.e., *ϕ* is incremented in 2° steps). Then *B*_av_ for each coil probe is evaluated and combined according to Eq. (1) for each value of *ϕ* to obtain *B*_av3_, *B*_av3_ is compared with *B*_0_, and the largest difference is saved. Because of the symmetry of the problem, a total rotation of 90° is required to cover all the cases (with 2° increments).
- The angle *α*_1_ is advanced in 5° steps and the above comparisons are repeated as the probe is rotated about the *z*‴-axis or ***n***_1_ direction. The maximum value of *α*_1_, without duplication of results is 90°.
- For each value of *α*_1_, *α*_2_ is incremented from 0° in steps of 5° and the above comparisons are repeated. Because of symmetry arguments, a total rotation of 180° is required to consider all the cases without duplication.
- Following the above calculations, different orientations of the magnetic dipole are considered by changing the angle *θ* in 15° steps and repeating steps (i) through (iii). The choices of increments indicated above were found to provide adequate sensitivity for determining Δ*B*_av3_.
- Steps (i) through (iv) are repeated for different values of *r.*

**Fig. 5:**
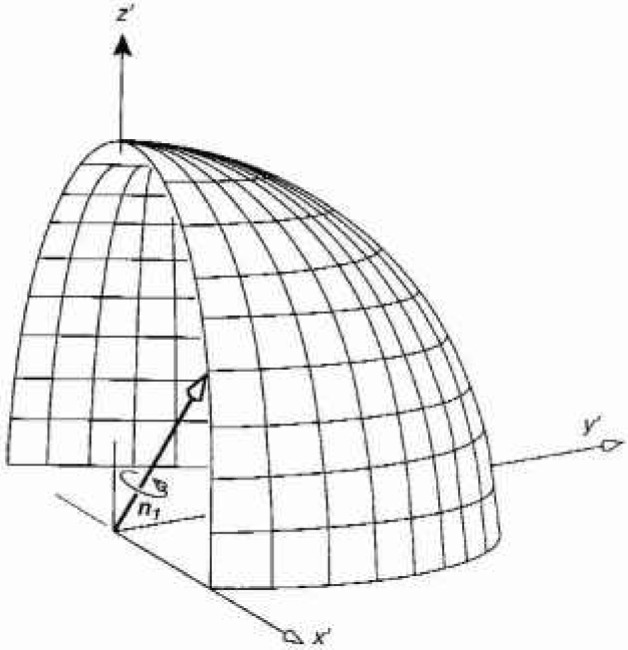
Trajectories of ***n***_1_ during search for Δ*B*_max3_. The unit vectors ***n****_2_* and ***n***_3_ are not shown but maintain their orthogonal relationship wilh ***n***_1_ throughout the search.

A diagram schematically indicating several positions for ***n***_1_, and rotations about ***n***_1_, as the above protocol was carried out for a fixed value of *r* is shown in Fig. 5.

## 4. Results and Discussion

As already noted, an earlier search for Δ*B*_max3_ [3] was not as comprehensive as the one described in this paper. While the ratio *r/a* and *θ* could be varied without restriction, the rotations of the three-axis probe were limited to simple rotations about the *x*′-,*y*′-, or *z*′-axis. That is, it was not possible to consider differences between *B*_av3_ and *B*_0_ for combinations of rotations about two or three axes. This problem has been overcome with a more generalized expression for *B*_av_ compared to the ones used in the earlier calculation. What is perhaps surprising, however, is that the Δ*B*_max3_ values obtained with the more comprehensive search protocol are the same as previously calculated. That is, Δ*B*_max3_ is negative and occurs for all *r/a* values when *θ* = 90°, as previously found, and correspond to the Δ*B*_max3_ values determined earlier following simple rotations about the *y*′-axis (referred to as "α rotations" in Ref. [3]). Numerical values of Δ*B*_max3_ are provided in Table 1 as a function of *r/a.*

## 5. Conclusions

The present calculations have determined the largest differences between the resultant magnetic field, *B*_av3_, and the field value at the center of the probe *b*_0_, assuming a dipole magnetic field. These largest differences, designated Δ*B*_max3_, are reported in Table 1 as a function of normalized distance, *r/a*, from the center of the dipole and agree with values previously found after a far less comprehensive search [3]. The quantity, Δ*B*_max3_, can be regarded as the largest error due to instrumental averaging effects. As noted earlier, because the relative orientations of the dipole and three-axis probe are not known for a given *r/a* under typical measurement conditions, there will be a range of possible differences between *B*_av3_ and *B*_0_. Thus, ideally, it would be desirable to determine the distribution of differences between *B*_av3_ and *B*_0_ and treat the problem using a statistical approach, but that has been left to a future calculation.

Because the dipole field is a good approximation of fields produced by many electrical appliances, the information in Table 1 should be taken into account when total uncertainties are being determined during measurements of magnetic fields from appliances. For example, if the resultant magnetic field is to be measured at a distance *r* from an appliance with a combined relative standard uncertainty [5] of less than ± 10%, magnetic field meters with three-axis probes having radii *a* such that *r/a* ≈ 3 should be considered unsuitable. Three-axis probes having radii such that *r/a* =5 would conservatively be considered suitable if the combined relative standard uncertainty from all other sources (e.g., calibration process, frequency response) amounted to about 3% or less, since 6.9% + 3.0% = 9.9%, where 6.9% is taken from Table 1 for *r/a* =5.

## Figures and Tables

**Table 1:** Values of Δ*B*_max3_ as a function of normalized distance *r/a* from magnetic dipole

*r/a*	Δ*B*_max3_(%)
3	−19.6
4	−10.8
5	−6.9
6	−4,8
7	−3.5
8	−2.7
9	−2.1
10	−1.7
11	−1.4
12	−1.2
13	−1.0
14	−0.9
15	−0.8

## References

[1] Mader DL, Peralta SB (1992). Residential Exposure to 60-Hz Magnetīc Fields From Appliances. Bioelectromagnetics.

[2] IEEE Magnetic Fields Task Force (1993). A Protocol for Spot Measurements of Residential Power Frequency Magnetic Fields. IEEE Trans Power Delivery.

[3] Misakian M (1993). Coil Probe Dimensions and Uncertainties During Measurements of Nonuniform ELF Magnetic Fields. J Res Natl Inst Stand Tcchnol.

[4] Corson D, Lorrain P (1962). Introduction to Electromagnetic Fields and Waves.

[5] Taylor BN, Kuyatt CE (1993). Guidelines for Evaluating and Expressing the Uncertainty of NIST Measurement Results.

